# Anesthetic effect of different doses of butorphanol in patients undergoing gastroscopy and colonoscopy

**DOI:** 10.1186/s12893-021-01262-8

**Published:** 2021-05-27

**Authors:** Shun Lv, Defeng Sun, Jinglin Li, Lin Yang, Zhongliang Sun, Yan Feng

**Affiliations:** 1 Department of Anesthesiology, The First Affiliated Hospital of Dalian Medical University, No. 5 Longbin Road, Dalian, 116011 China; 2 Department of Neuroelectrophysiology, The First Affiliated Hospital of Dalian Medical University, No. 222 Zhongshan Road, Dalian, 116011 China

**Keywords:** Pre-injection, Butorphanol, Gastroscopy with colonoscopy, Anesthetic effect

## Abstract

**Background:**

This study aimed to investigate the anesthetic effect of butorphanol with different doses in patients undergoing gastroscopy and colonoscopy.

**Methods:**

480 patients undergoing gastroscopy and colonoscopy were recruited and randomly divided into four groups to receive different doses of butorphanol (Group A = 2.5 μg/kg, Group B = 5 μg/kg, Group C = 7.5 μg/kg and Group D = 10 μg/kg). Butorphanol was administered 5 min before propofol infusion. The primary outcome was the incidence of body movement. Secondary outcomes were postoperative recovery time, length of stay in the Post-Anesthesia Care Unit (PACU), the total dose of propofol, and the incidence of intraoperative hypoxemia, propofol injection pain, cough, postoperative nausea and vomiting, drowsiness, and dizziness.

**Results:**

The incidence of body movement and the dose of propofol in Group C and D were lower than those in Group A and B (*P* < 0.05). The incidence and intensity of propofol injection pain and the incidence of cough in Group B, C, and D were lower than those in Group A (*P* < 0.05). The length of stay in PACU and the incidence of postoperative drowsiness and dizziness were higher in Group D than in Group A, B, and C (*P* < 0.05).

**Conclusion:**

Intravenous pre-injection of 7.5 μg/kg butorphanol with propofol can be the optimal dosage for patients undergoing gastroscopy and colonoscopy.

*Trial registration:* Trial registration: Chinese Clinical Trial Registry, ChiCTR2000031506. Registered 3 April 2020—Retrospectively registered, http://www.medresman.org.cn.

**Supplementary Information:**

The online version contains supplementary material available at 10.1186/s12893-021-01262-8.

## Background

Gastrointestinal endoscopy is the most reliable method for the diagnosis of gastrointestinal cancer [1]. However, many patients are reluctant to undergo endoscopy due to the associated side effects like nausea, retching, visceral pain, and other discomforts during the procedure. Some patients also experience preoperative tension and anxiety [2]. Nonetheless, with the development of efficient diagnosis and treatment methods, patients are willing to opt for gastrointestinal endoscopy under anesthesia/sedation [3]. Currently, the most used anesthesia for painless gastrointestinal endoscopy is intravenous propofol combined with a small concentration of opioids [4]. However, sometimes the inappropriate treatment of propofol leads to injection pain, affecting patient satisfaction and the post-anesthesia discharge score (PADS) [5–7].

Butorphanol tartrate, a mixed agonist and antagonist of the opioid receptor, produces an analgesic effect by agonizing the κ receptor. The increased frequency of spontaneous opening of the pyloric sphincter induced by butorphanol tartrate makes endoscopy easier and faster to perform [8, 9]. It partially antagonizes the mu opiate (μ) receptor, thereby reducing the incidence of postoperative adverse effects such as PONV. Due to the lighter degree of respiratory depression, butorphanol tartrate has been widely used in the anesthesia for patients undergoing gastrointestinal endoscopy in recent years [10]. However, the sedation caused by butorphanol tartrate could also lead to dizziness, lethargy, and other adverse reactions in the recovery period, which affects the discharge score and reduces the diagnosis and treatment efficiency [11].

Previous studies showed that the incidence of dizziness and drowsiness induced by butorphanol tartrate is dose-dependent [12, 13]. Butorphanol has been widely used to control perioperative pain. However, the ideal dose of butorphanol for gastroscopy with colonoscopy has not been investigated. This study aimed to explore the optimal dosage of butorphanol tartrate in patients undergoing gastroscopy with colonoscopy.

## Methods

This prospective experimental study was carried out at the Endoscopic Center of Jinpu Hospital, Dalian Medical University, between March and September 2019. Following the approval of the Medical Ethics Committee (code: PJ-KS-KY-2019-28) and was registered at the Chinese Clinical Trial Center (Registration Number: ChiCTR2000031506). All methods were performed in accordance with the ethical guidelines and regulations. This study was carried out in compliance with the CONSORT guidelines. Patients undergoing painless gastrointestinal endoscopy with colonoscopy, aged between 25 and 64 years, and American Society of Anesthesiologists (ASA) grading between I-III, without cardiovascular, respiratory, liver, renal diseases, and impaired cognitive functions were included. Those with a history of drug use, recent use of sedatives, anti-depressants, or analgesics were removed from this research. Written informed consent was signed by the participants before the enrollment.

### Sample size calculation

The sample size was calculated on a website (https://clincalc.com/stats/samplesize.aspx). Based on our pre-trial test, the incidence of body movement was 50% in the control group, and that in the butorphanol tartrate group was ~ 10–30%. The sample size was calculated by using the power level method. The power was set to 0.8, and α was set to 0.05. A total of 372 patients were estimated to be included for analysis (93 in each group) to achieve a power of 80%. Considering a possible dropout rate of 20%, a total of 480 patients were estimated to be included in the study.

### Study groups

Four hundred eighty participants were randomly allocated in four groups by a computerized software (R); A, B, C, and D (n = 120 in each group), to be receiving 2.5 μg/kg, 5 μg/kg, 7.5 μg/kg, and 10 μg/kg of butorphanol respectively. Butorphanol (Trade Name: Nuoyang, 2 ml: 4 mg, Jiangsu Hengrui Medicine Co., Ltd. Batch Number: 190315BP. National Pharmaceutical Standard: H20143106) was injected 5 min before the endoscopic surgery by a nurse, who did not participate in the following anesthesia and evaluation procedures.

### Anesthesia

Patients in each group were kept nil by food for 8 h and water for 2 h before the endoscopic procedure. Vein access was established on the posterior right hand. 500 mL Ringer's solution and 0.3 mg Ramosetron were administrated intravenously (i.v.). The patients were monitored for electrocardiogram, non-invasive blood pressure, and pulse oximetry. Following this, different doses of butorphanol were administrated IV in all the patients, as described previously by a senior anesthesiologist.

Anesthesia was induced intravenously 2 mg/kg 1% propofol in one minute. Endoscopy was initiated after the loss of eyelash reflex. Propofol was maintained at a rate of 4 mg/kg/h to maintain an appropriate depth of anesthesia during the endoscopy, and 0.5 mg/kg of propofol was added if body movements occurred during the procedure. During anesthesia, the patients were routinely monitored for electrocardiogram, non-invasive blood pressure, and pulse oximetry. Oxygen was inhaled through a mask, and the oxygen flow rate was 6–8 L/min. The anesthesiologist closely observed the thoracic movement changes, respiratory rate, and sedation depth. The procedure was temporarily paused if the heart rate (HR) reached < 40 beats/minute or increased to > 100 beats/minute. If the symptom was not relieved, 0.4 mg atropine (i.v.) was administered. If the anesthesia depth was not maintained, 0.5–1 mg/kg propofol was injected IV; otherwise, 5–10 mg esmolol was administered i.v. Likewise, when systolic blood pressure (SBP) reached ≥ 180 mmHg and/or diastolic blood pressure (DBP) reached ≥ 100 mmHg, 5–10 mg urapidil was administered IV. When the systolic blood pressure dropped to ≤ 80 mmHg, 6 mg ephedrine was administered i.v.

Polyps under endoscopy were also operated if required, and the sedation level of patients was assessed by the anesthesiologist as per the Modified Observer's Assessment of Alertness/Sedation (MOAA/S) Scale (Additional file 1: Table S1) [14]. If the score was ≤ 3 points, the intravenous injection rate of propofol was maintained; if the score was > 3 points, 0.5 mg/kg propofol was added to IV propofol. Propofol infusion was stopped when the endoscopy finished. The incidence and severity of injection pain were evaluated according to the Pain Scores of 4-point Verbal Rating Scale (Additional file 1: Table S2).

After the endoscopic procedure, the patient was transferred to the Post-Anesthesia Care Unit (PACU) for further observation. The degree of recovery was evaluated using the MOAA/S Scale every 3 min. If the MOAA/S score was > 3 points, the patient was re-evaluated every 1 min until the patient's MOAA/S score reached 5 (indicating that the patient has recovered). A Visual Vertigo Analogue Scale was used to assess the severity of dizziness before the patients left the PACU. Only those with a PADS ≥ 9 points (Additional file 1: Table S3) [15] and a vertigo score < 7 were discharged from the hospital.

### Observation indexes

General information such as age, gender, weight, ASA grade, underlying diseases, and the conditions of removing polyps were recorded. The mean arterial pressure (MAP), HR, and pulse oxygen saturation (SpO_2_) of the patients in all the groups were recorded at these time points: before anesthesia (T0), scope passing through the oropharynx (T1), scope passing through the sigmoid colon (T2), scope passing through the liver area (T3), the procedure finished (T4), and when the patients gained consciousness (T5). Primary outcomes were evaluated in terms of the incidence of body movement. Similarly, secondary outcomes were evaluated in terms of postoperative recovery time, PACU retention time, integral dose of propofol, the incidence of intraoperative hypoxemia, injection pain, cough, postoperative nausea and vomiting, and postoperative drowsiness and dizziness.

#### Operational definitions

- Body movement was defined as the twisting of the patient’s body due to the stimulation of the endoscopy, making it difficult to proceed with the procedure without additional propofol.
- Hypoxemia was defined as blood oxygen saturation ≤ 90% and the duration lasting for more than a minute or the use of any airway interventions (mandibular lift, oxygen flow, mechanical ventilation, etc.) during the endoscopy.
- Recovery time was set as the time from discontinuing propofol to improving the MOAA/S score to five points. The delayed recovery was defined as the patient's MOAA/S score ≤ 4 points continuing for > 60 min after discontinuing anesthesia.
- Drowsiness was defined as a response to slight probing and verbal stimuli and falling asleep after the stimuli were stopped.

### Statistical analysis

SPSS 26.0 (IBM, USA) was used for the statistical analysis of data. The numerical variables were expressed as mean ± standard deviation (SD). The categorical variables were expressed as rate. Data that did not meet the normal distribution were expressed in the median (quartile). One-way analysis of variance was used to analyze normal data, whereas the Kruskal–Wallis test was performed for data that was not normal. Categorical variables were analyzed by Pearson’s chi-square test. A P-value of less than 0.05 was statistically significant. The results were analyzed by Per Protocol analysis to eliminate the influence of various biases.

## Results

**Fig. 1:**
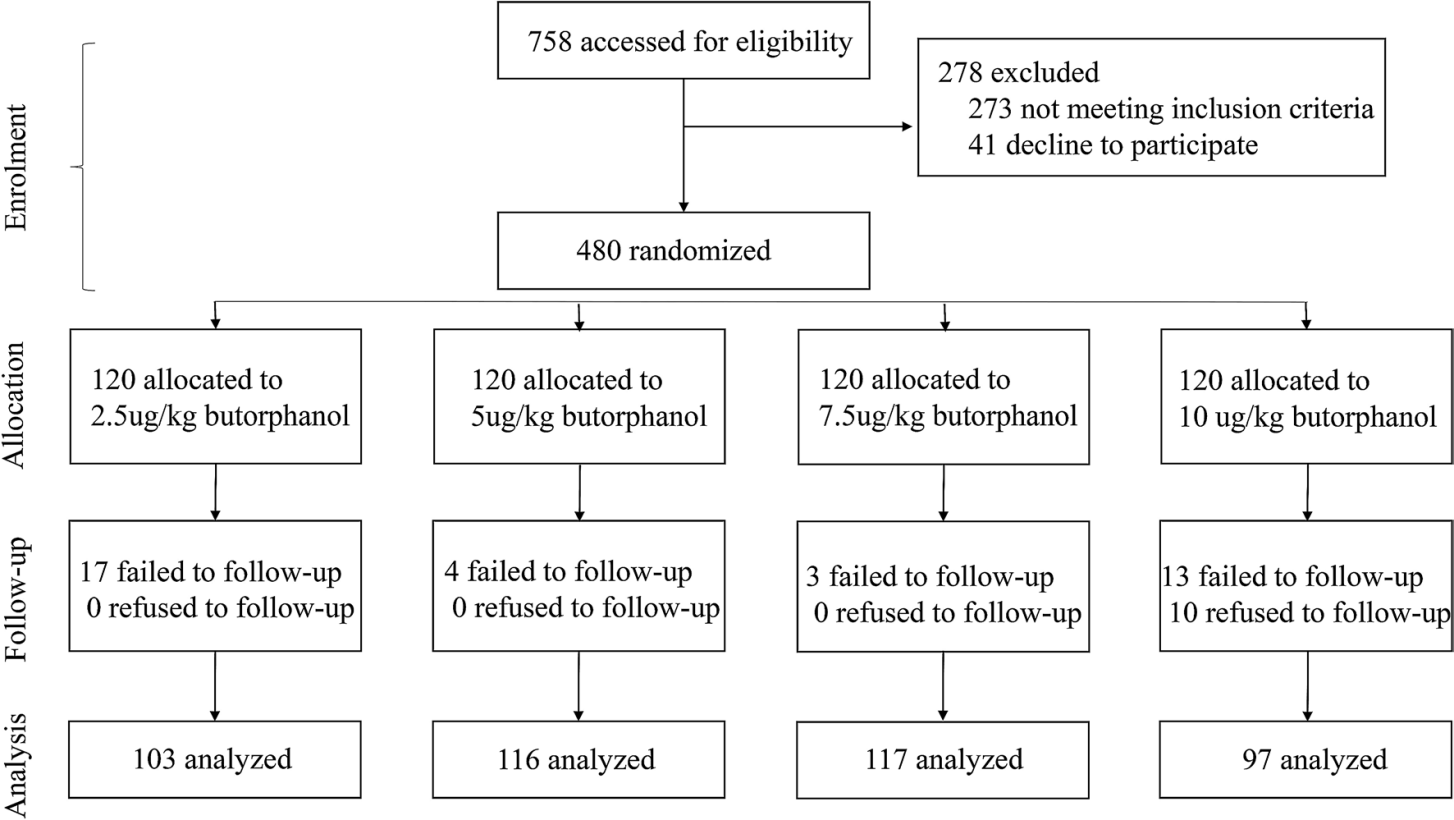
Flow chart of the clinic trial, depicting the distribution of recruited patients

A total of 758 patients were part of the study in the beginning. After the initial assessment, 273 patients did not meet the inclusion criteria, and 41 patients declined to participate in the study. During the process, 47 patients discontinued follow-up and were hence excluded. A total of 433 patients from all the groups completed the study (Fig. 1).

The demographic variables such as age, gender, weight, ASA grade, the combined underlying diseases, and the removal of polyps during the endoscopy among groups were not different between these groups (*P* > 0.05), indicating that homogeneity was maintained (Table 1).

**Table 1:** Sociodemographic and clinical features of patients in four groups (N = 433)

	Group A (N = 103)	Group B (N = 116)	Group C (N = 117)	Group D (N = 97)	*F / χ *^2^	*P*
Age (year)	50 (41–55)	52 (41–58)	48 (42–55)	51 (43–58)	2.029	0.566
Male (%)	53 (51.5)	66 (56.9)	58 (49.6)	52 (53.6)	1.369	0.713
Weight (kg)	68 (61–75)	69 (64–75)	66 (60–74)	67 (60–73)	5.606	0.132
ASA grade (I/II/III)	73/29/1	75/40/1	74/42/1	75/20/1	7.847	0.250
Combined diabetes or hypertension (%)	9 (8.7)	12 (10.3)	8 (6.8)	7 (8.2)	2.836	0.970
Removal of polyps (%)	10 (9.7)	12 (10.3)	10 (8.5)	12 (12.4)	0.880	0.830

The data are presented as the median and interquartile range (M (P25, P75) or mean ± standard deviation or numbers of patients. Group A: Butorphanol 2.5 μg/kg group; Group B: Butorphanol 5 μg/kg group; Group C: Butorphanol 7.5 μg/kg group; Group D: Butorphanol 10 μg/kg group;* P*:* P* value;* F*/*χ*^2^:* F* value or *χ*^2^

The incidence of body movement and dosage of propofol administered in groups C (11%) and D (8%) were much lower than those in groups A (42%) and B (33%) (*χ *^2^ = 45.902, *F* = 38.020, *P* < 0.05) (Table 2). The PACU time of Group D was 14 min, which was longer than that of groups A (11 min), B (11 min), and C (12 min) (*F* = 26.040, *P* < 0.05). The recovery times were not significantly different among the four groups (*F* = 4.521, *P* > 0.05) (Table 2).

**Table 2:** Comparison of the incidence of body movement, amount of Propofol, recovery time, and PACU time in the four groups

	Group A (N = 103)	Group B (N = 116)	Group C (N = 117)	Group D (N = 97)	*F / χ *^2^	*P*
Incidence of body movement (%)	42 (40.8)	33 (28.4)	11 (9.4) *^‡^	8 (8.2) *^‡^	45.902	< 0.001
Amount of Propofol (mg)	220 (188–255)	200 (182–225)	192 (162–220)†#	185 (165–205)^‡^*	38.020	< 0.001
Recovery time (min)	6 (5–8)	6 (5–8)	6 (4–9)	5 (4–8)	4.521	0.210
PACU time (min)	11 (9–14)	11 (8–13)	12 (10–15)	14 (11–17)*^‡§^	26.040	< 0.001

The data are presented as the median and interquartile range (M (P25, P75). Group A: Butorphanol 2.5 μg/kg group; Group B: Butorphanol 5 μg/kg group; Group C: Butorphanol 7.5 μg/kg group; Group D: Butorphanol 10 μg/kg group

^†^Means compared with Group A (*P* < 0.05); ^‡^means compared with Group A (*P* < 0.001); #means compared with Group B (*P* < 0.05); *means compared with Group B (*P* < 0.001); §means compared with Group C (*P* < 0.001)

The incidence of injection pain by propofol in Group D was 19%, which was significantly lower in groups A (61%), B (45%), C (36%), and (*χ *^2^ = 57.303, *P* < 0.05). The incidence of mild pain in Group D was also significantly lower than in groups A and B (*P* < 0.05). There was no significant difference in the incidence of hypoxemia and PONV among the four groups of patients (*χ *^2^ = 5.491, *P* > 0.05). The incidence of cough in the three groups B, C, and D was significantly lower than in Group A (*χ *^2^ = 19.647, *P* < 0.05). The incidence of dizziness and drowsiness in group D (64% and 13%) was significantly higher than that in groups A (38% and 3%), B (38% and 4%), and C (56% and 5%) (*χ *^2^ = 36.385, *P* < 0.05) (Table 3).

**Table 3:** Comparison of complications during and after the endoscopy in the four groups

		Group A (N = 103)	Group B (N = 116)	Group C (N = 117)	Group D (N = 97)	*F / χ*^2^	*P*
Propofol injection pain (%)	Yes	61 (59.2)	45 (38.8)*	36 (30.8)*	19 (19.6)*^#^	57.303	** < 0.001**
	Mild	35 (34.0)	38 (32.8)	32 (27.4)	15 (15.5)*^#^		
	Moderate	26 (25.2)	7 (6.0)*	4 (3.4)*	4 (4.1)*		
Hypoxemia (%)	Yes	12 (11.7)	9 (7.8)	12 (10.3)	8 (8.2)	1.221	0.748
Cough (%)	Yes	23 (22.3)	9 (7.8)*	8 (6.8)*	6 (6.2)*	19.647	** < 0.001**
PONV (%)	Yes	0 (0.0)	2 (1.7)	0 (0.0)	0.0 (0.0)	5.491	0.139
Dizziness (%)	Yes	38 (36.9)	38 (32.8)	56 (47.8)	64 (66.0)*^#**†**^	36.385	** < 0.001**
Drowsiness (%)	Yes	3 (2.9)	4 (3.4)	5 (4.3)	13 (13.4)*^#**†**^	13.562	**0.004**

Qualitative data were presented as (%).Group A: Butorphanol 2.5 μg/kg group; Group B: Butorphanol 5 μg/kg group; Group C: Butorphanol 7.5 μg/kg group; Group D: Butorphanol 10 μg/kg group. *Means compared with Group A (*P* < 0.05); # means compared with Group B (*P* < 0.05); **†**means compared with Group C (*P* < 0.001)

**Fig. 2:**
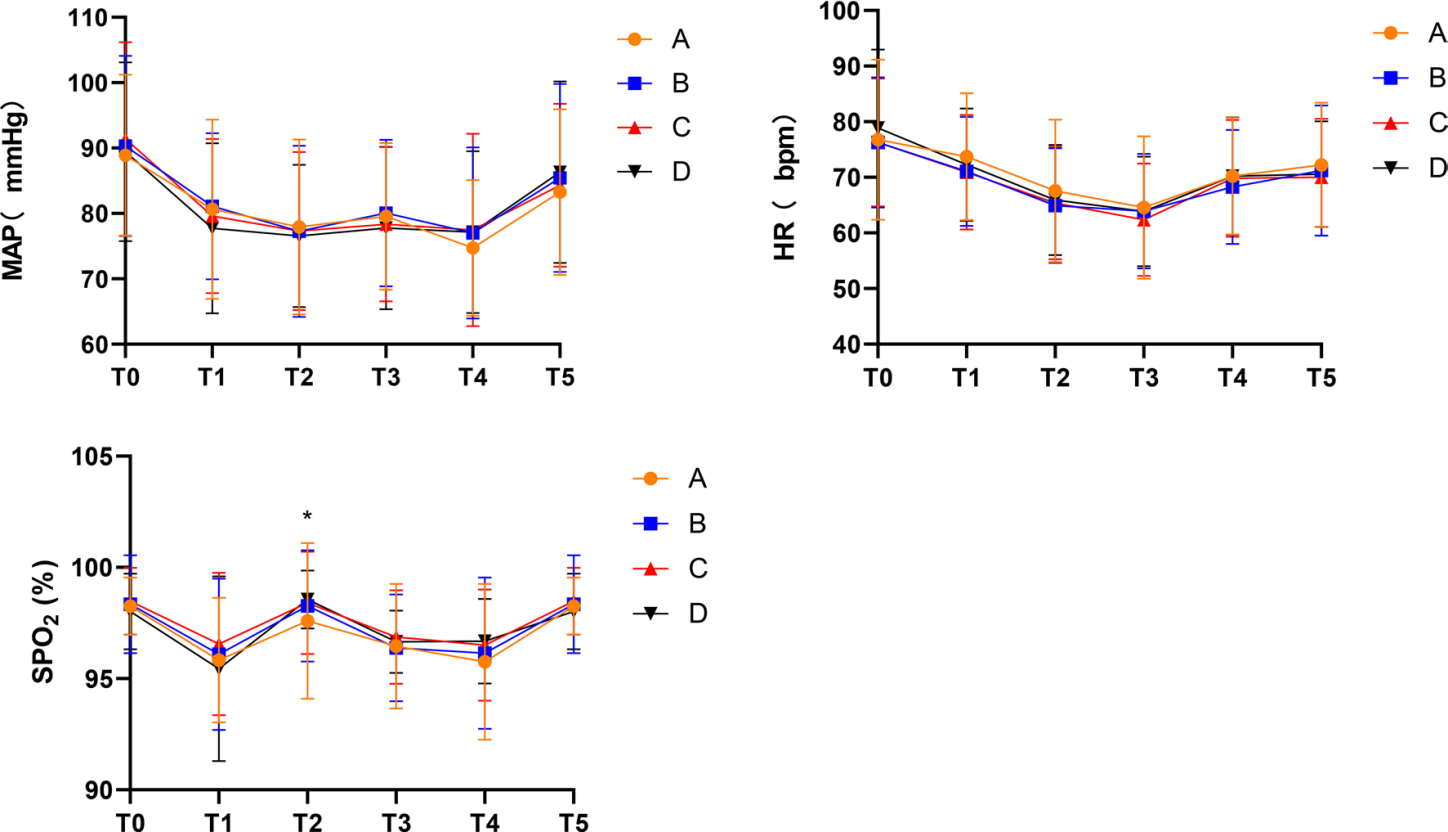
The change of MAP, HR, and SpO2 in the four groups

MAP and HR were not significantly different between the four groups of patients during the endoscopy (*P* > 0.05). However, the SpO_2_ of group A after colonoscopy got through the sigmoid colon (T2) was lower than that in groups B, C, and D (*F* = 12.809, *P* < 0.05) (Fig. 2).

## Discussion

In this study, the incidence of body movements and the dosage of propofol in groups C and D were lower than in groups A and B (*χ *^2^ = 45.902, *F* = 38.020, *P* < 0.05). The incidence of cough in groups B, C, and D was significantly lower than in group A (*χ *^2^ = 19.647, *P* < 0.05), indicating the protective role of butorphanol. In combination with propofol, butorphanol could achieve a better anesthetic effect during gastrointestinal endoscopy. The incidence of body movement, cough, and the required dosage of propofol was significantly reduced, which helps avoid the increased risk of complications such as respiratory depression due to a high dose of propofol.

Li and colleagues found that the incidence of hypoxemia was 15% when propofol alone was administered for upper gastrointestinal endoscopy under the most suitable infusion rate [16, 17]. However, in our study, hypoxemia incidence was only between 8.2 and 11.7%, significantly lower than the results reported by Li and co-workers. This may be linked to the administration of butorphanol and lowering the dosage of propofol. This suggests that in gastrointestinal endoscopy, butorphanol combined with propofol could reduce the incidence of hypoxemia. The lower pulse oximetry of group A at T2 is associated with the high incidence of body movement due to less butorphanol in group A.

Agarwal and colleagues found that using 2 mg butorphanol for preemptive analgesia effectively reduced propofol injection pain than intravenous lidocaine [18]. In this study, the incidence of propofol injection pain in groups B, C, and D was significantly lower than that in group A (*χ * = 57.303, *P* < 0.05), and the incidence of mild pain in group D was also significantly lower than that in group B (*P* < 0.05), indicating that prophylactic intravenous injection of 5–10 μg/kg butorphanol 10 min before the operation can effectively reduce the incidence of propofol injection pain. The highest concentration, i.e., 10 μg/kg butorphanol, showed the maximum analgesic effect, which is compatible with the previous study that the ED95 of butorphanol for the anesthesia of gastrointestinal endoscopy was 9.07 μg/kg [19]. As an agonist of the kappa receptor, butorphanol is suggested to produce a certain degree of sedation, which could lead to adverse reactions such as dizziness and drowsiness during the recovery period, affected the discharge score, and reduced the diagnostic and treatment efficiency. Previous studies have suggested a dose-dependence of butorphanol in the incidence of postoperative dizziness and drowsiness [11, 12].

The time in PACU was also significantly longer in group D than in groups A, B, and C, which may be related to the increased incidence of dizziness and drowsiness due to a higher dose of 10 μg/kg butorphanol. The young and middle-aged patients were more sensitive to the feeling of dizziness, which made this study particularly practical.

The main limitation of this study is that we only observed the anesthetic effect of butorphanol combined with propofol. So a placebo/control group (without butorphanol) was not included. Another major limitation of our study is that we don’t compare the satisfaction of patients and doctors. Finally, the incidence of dizziness, nausea, and vomiting, pain should have been followed up for better clarity on the long-term effects of butorphanol.

## Conclusion

7.5 μg/kg of intravenous butorphanol pre-injection 5 min before the procedure combined with propofol can safely and effectively be used to induce anesthesia in patients undergoing painless gastroscopy combined with colonoscopy. It has the advantage of suppressing propofol injection pain and decreases the incidence of dizziness and drowsiness.

## Supplementary Information

**Additional file 1: Table S1.** MOAA/S (Modified Observer’s Assessment of Alertness/ Sedation) Scale. **Table S2.** Pain Scores of 4-point Verbal Rating Scale. **Table S3.** Post Anesthesia Discharge Score (PADS).

## Data Availability

The datasets used and/or analyzed during the current study are available from the corresponding author on reasonable request.

## Abbreviations

PACU: Post-Anesthesia Care Unit
PONV: Postoperative nausea and vomiting
PADS: Post-anesthesia discharge score
ASA: American Society of Anesthesiologists
STAI-S: State-trait anxiety inventory state
SBP: Systolic blood pressure
DBP: Diastolic blood pressure
MOAA/S: Modified observer's assessment of alertness/sedation
SD: Standard deviation
