# Supplementary material for: Anesthetic effect of different doses of butorphanol in patients undergoing gastroscopy and colonoscopy
Source: BMC Surg. 2021 May 27;21:266. doi: 10.1186/s12893-021-01262-8 (PMC8161954; doi:10.1186/s12893-021-01262-8)
Supplement: Supplementary file 1 — Additional file 1: Table S1. MOAA/S (Modified Observer’s Assessment of Alertness/ Sedation) Scale. Table S2. Pain Scores of 4-point Verbal Rating Scale. Table S3. Post Anesthesia Discharge Score (PADS). [file 12893_2021_1262_MOESM1_ESM.docx]

**Anesthetic effect of different doses of butorphanol in patients undergoing gastroscopy and colonoscopy**

**Short title:** Butorphanol in gastroendoscopy and coloscopy

Shun Lv^1^, Defeng Sun ^1,*^, Jinglin Li ^1^, Lin Yang^2,*^, Zhongliang Sun^1^, Yan Feng^1^

^1^Department of anesthesiology, The First Affiliated Hospital of Dalian Medical University, Dalian, 116011, China.

^2^Department of Neuroelectrophysiology, The First Affiliated Hospital of Dalian Medical University, Dalian, 116011, China.

***Corresponding author:**

Defeng Sun, No. 5 Longbin Road, The First Affiliated Hospital of Dalian Medical University, Dalian, 116011, China.

Email: sundefengyl@163.com

Tel: +8618098876191

Lin Yang, No.222 Zhongshan Road, The First Affiliated Hospital of Dalian Medical University, Dalian, 116011, China.

Email: sdf-yl@163.com

Tel: +8618098876288

**Table S1. MOAA/S (Modified Observer’s Assessment of Alertness/ Sedation) Scale**

| Score | Response to external stimulus |
| --- | --- |
| 5  4  3  2  1  0 | Responds readily to name spoken in normal tone  Lethargic response to name spoken in normal tone  Responds only after name is called loudly and/or repeatedly  Responds only after mild prodding or shaking  Does not respond to mild prodding or shaking  Does not respond to painful stimulus |

The full version can be obtained from the following website: [www.giejournal.org](http://www.giejournal.org).

**Table S2. Pain Scores of 4-point Verbal Rating Scale**

| Score of pain | Severity of pain | Response |
| --- | --- | --- |
| 0 | None | No pain reported in response to questioning |
| 1 | Mild | Pain reported in response to questioning only without any behavior signs |
| 2 | Moderate | Pain reported in response to questioning and accompanied by a behavioral sign or pain reported spontaneously without questioning |
| 3 | Severe | Strong verbal response or response accompanied by facial grimacing, arm withdrawal, or tears. |

**Table S3.** **Post Anesthesia Discharge Score (PADS)**

| **Category** | **Status** | **Score** |
| --- | --- | --- |
| Vital signs (BP and pulse) | Within 20% of preoperative baseline | 2 |
|  | 20-40% of preoperative baseline | 1 |
|  | > 40% of preoperative baseline | 0 |
| Activity | Steady gait, no dizziness | 2 |
|  | Requires assistance | 1 |
|  | Unable to ambulate | 0 |
| Nausea and vomiting | Mild, no need of medication | 2 |
|  | Moderate, response to medication | 1 |
|  | Severe, no response to medication | 0 |
| Pain | Mild | 2 |
|  | Moderate | 1 |
|  | Severe | 0 |
| Surgical bleeding | Mild | 2 |
|  | Moderate | 1 |
|  | Severe | 0 |
